# Spatial patterns of laboratory-confirmed leptospirosis in north-eastern Peninsular Malaysia, 2016-2023

**DOI:** 10.4178/epih.e2025030

**Published:** 2025-05-29

**Authors:** Hazlienor Mohd Hatta, Kamarul Imran Musa, Nik Mohd Hafiz Mohd Fuzi, Paula Moraga

**Affiliations:** 1 Department of Community Medicine, School of Medical Sciences, Universiti Sains Malaysia, Kota Bharu, Malaysia; 2 Communicable Disease Control Unit, Disease Control Section, Kelantan State Health Department, Kota Bharu, Malaysia; 3 Computer, Electrical and Mathematical Sciences and Engineering Division, King Abdullah University of Science and Technology (KAUST), Thuwal, Saudi Arabia

**Keywords:** Communicable diseases, Epidemiology, Leptospirosis, Spatial analysis

## Abstract

**OBJECTIVES:**

Leptospirosis presents significant public health challenges in endemic regions such as north-eastern Peninsular Malaysia. Spatial analysis is essential for visualising disease incidence and distribution, assessing vulnerability based on geographical and socioeconomic factors, and ultimately informing targeted interventions, optimising resource allocation, and enhancing surveillance strategies. This study aimed to determine the incidence and characterise the spatial distribution of leptospirosis in Kelantan, Malaysia.

**METHODS:**

All laboratory-confirmed leptospirosis cases reported in Kelantan between 2016 and 2023 were extracted from the Communicable Disease Control Information System e-Notifikasi online database. Spatial analyses were performed using the spatstat, spdep, and ggplot2 packages within the RStudio integrated development environment.

**RESULTS:**

The analysis encompassed 1,534 laboratory-confirmed leptospirosis cases. The average crude annual incidence of leptospirosis cases per 1,000 population from 2016 to 2023 was 0.101 (95% confidence interval, 0.038 to 0.164). Incidence varied considerably across districts and subdistricts, initially higher in the north but declining over time, while consistently high and increasing incidence was observed in the southern region. Significant clustering of leptospirosis cases occurred throughout the studied years, except during the coronavirus disease 2019 pandemic. Hotspots were initially prevalent in northern areas but later emerged in south-eastern and southern regions. Significant spatial autocorrelation evolved from high-low to high-high clusters, particularly evident in central and southern regions.

**CONCLUSIONS:**

This study provides valuable local epidemiological and spatial insights into the endemicity of leptospirosis. The findings highlight the need for targeted interventions and continued surveillance to effectively mitigate the leptospirosis burden in endemic areas.

## GRAPHICAL ABSTRACT

[Fig f6-epih-47-e2025030]

## Key Message

• This study examined leptospirosis patterns in Kelantan, Malaysia, from 2016 to 2023, highlighting distinct regional variations in disease incidence.

• Case densities were high in the northern region, whereas incidence demonstrated higher risk in the central and southern regions.

• The study underscores the importance of considering both geographic location and population dynamics when planning interventions and allocating resources for disease control.

• The evident spatial clustering highlights the need for targeted public health interventions.

## INTRODUCTION

There is increasing concern regarding emerging and re-emerging infectious diseases, which commonly cause undifferentiated febrile illnesses that significantly contribute to morbidity and mortality among children and adults [1]. Common recent causes of febrile illness in tropical and subtropical regions include dengue, scrub typhus, leptospirosis, and enteric fever [2]. Among bacterial aetiologies, leptospirosis is gaining importance due to its frequent association with severe complications and higher mortality [3]. Leptospirosis is a re-emerging zoonosis caused by Gram-negative spirochaete bacteria belonging to the genus *Leptospira* within the family *Leptospira*ceae. It exhibits substantial genetic diversity, with 38 pathogenic *Leptospira* species identified to date [4]. Animals, especially rats, are the primary reservoirs, often remaining asymptomatic carriers with colonisation of renal tubules and urinary excretion of *Leptospira* into the environment [5]. Humans contract leptospirosis through direct contact with infected animal urine, reproductive fluids, blood, or tissues, or more commonly, through exposure to contaminated environments, including ingestion of contaminated food or water [5]. Upon contact with contaminated water, *Leptospira* can enter the human body through skin lesions or mucous membranes of the eyes, mouth, or nose [5].

Globally, leptospirosis is estimated to cause approximately 1.03 million to 1.75 million cases and nearly 59,000 deaths annually. The highest incidences occur in South and Southeast Asia, Oceania, the Caribbean, Andean, Central and Tropical Latin America, and East sub-Saharan Africa, regions where the disease is also frequently reported among travellers [6]. In Malaysia, leptospirosis is a mandatory notifiable disease. Its incidence has increased notably from 1.03 cases per 100,000 population in 2004 to 30.2 cases per 100,000 in 2015 [7], with particularly high incidences reported in states including Kelantan, Selangor, Sarawak, Kedah, and Terengganu [8]. Malaysia experienced a sharp rise in leptospirosis cases over the past decade, with reported cases increasing from 1976 (with 69 deaths) in 2010 to 7,806 cases in 2014, before gradually decreasing [9].

Kelantan, located in north-eastern Peninsular Malaysia, remains highly endemic for leptospirosis, significantly contributing to the state’s infectious disease burden, morbidity, and mortality. However, precise areas of endemicity remain inadequately defined [10]. Infectious diseases often exhibit distinct geographical distributions heavily influenced by socioeconomic, environmental, and bio-climatic factors. Traditional epidemiological methods are insufficient to adequately capture the spatial heterogeneity of leptospirosis due to these complex geographical and environmental interactions. Spatial analysis and clustering methods provide robust tools for visualising disease distribution, analysing localised transmission patterns, and identifying high-risk areas. Understanding the spatial distribution of leptospirosis is essential for guiding targeted public health initiatives, improving surveillance systems, and predicting outbreaks, thereby enabling more effective disease control. This study aimed to determine the incidence and characterise the spatial distribution of leptospirosis in Kelantan, Malaysia, with particular emphasis on endemicity and disease burden at the subdistrict level.

## MATERIALS AND METHODS

### Study area

The study was conducted in Kelantan, Malaysia, from October 2022 to March 2024. Kelantan, located in north-eastern Malaysia, is bordered by Thailand to the north and the South China Sea to the east. It covers approximately 15,104 square kilometres, representing about 4.5% of Malaysia’s total area. The state comprises 10 districts divided into 66 administrative subdistricts [11] (Supplementary Material 1). Kelantan has the highest average household size in Malaysia at 4.9 people per household, with a population density of 119 individuals per km^2^ [11] (Supplementary Material 2). Approximately 44.1% of the population resides in urban areas, predominantly in the northern region [12].

### Data collection

Data on confirmed leptospirosis cases were retrieved from Malaysia’s Communicable Disease Control Information System e-Notifikasi system, a passive surveillance platform for notifiable diseases. Cases were included if they were laboratory-confirmed from Kelantan using microscopic agglutination test (MAT; ≥1:400 titre or a 4-fold rise in paired sera), polymerase chain reaction, bacterial culture, or immunohistochemistry [13]. Each case was geolocated using handheld GPS devices with coordinates recorded in the WGS84 coordinate reference system (CRS). Coordinates were collected at the nearest accessible point in front of each case’s residence by trained district health officers within 1 week of notification. Population data for Kelantan’s subdistricts were sourced from the Department of Statistics Malaysia (DOSM). Geospatial data containing subdistrict boundaries were obtained as a shapefile using the Kertau Rectified Skewed Orthomorphic (RSO) Malaya meter projected CRS, commonly applied in Malaysia.

### Statistical analysis

Analyses were carried out using the R version 4.2.3 integrated development environment and RStudio software for Windows [14,15]. Case coordinates were converted to the RSO Malaya CRS (in metres) using the dplyr package [16]. Point counts within each subdistrict polygon were used to calculate crude incidence rates, and the 95% confidence intervals (CIs) were estimated using the Wilson score method [17]. Both point-pattern and polygon-based analyses were utilised due to their complementary roles in understanding leptospirosis distribution at different spatial scales. Point-pattern analysis identifies small-scale variations potentially overlooked in aggregated data, facilitating the detection of localised clusters linked to environmental or socio-demographic risk factors. Polygon-based analysis, using incidence data, accounts for the population at risk and enables regional comparisons, which are essential for district-level interventions. Integrating both approaches provides comprehensive insights into leptospirosis distribution, capturing local patterns and broader regional trends to inform targeted prevention and control efforts.

#### Point pattern analysis

Leptospirosis case density was estimated using kernel density estimation (KDE) from the spatstat package [18]; expressed as:


$$\hat{p_{n}}(x)=\frac{1}{nh^{d}}\sum_{i=1}^{n}K\;(\frac{x-X_{i}}{h})$$

Where *K* is the Gaussian smoothed kernel function and *h* is the smoothing bandwidth [19,20]. Multiple bandwidth selectors, including likelihood cross-validation (LCV), Cronie and van Lieshout’s criterion, Scott’s rule of thumb, and fixed sigma values at 5 km, 10 km, and 20 km, were tested [19,20]. The LCV method was ultimately selected because it provided an optimal balance between spatial detail and smoothing by minimising the estimated error function, thus avoiding over-smoothing and under-smoothing artefacts [19,21].

Spatial clustering of leptospirosis cases was assessed using average nearest neighbour distance analysis via the nearest neighbour index (NNI) and the $\hat{G}$-function from the spdep package. NNI was calculated as the ratio of the observed mean nearest neighbour distance to the expected distance under complete spatial randomness (CSR). An NNI value <1 indicates clustering, while a value >1 suggests dispersion [22]. A Z-score in NNI analysis measured how significantly the observed spatial pattern deviated from randomness, with negative values suggesting clustering and positive values indicating dispersion [22]. The $\hat{G}$-function measured the probability that the nearest neighbouring cases were found within a given distance *r* (in metres) based on the following formula:


$$\hat{G}(x)=\frac{r_{ik}\leq x,\;\forall i}{n}$$

Where *r_ik_* is the distance from the event at *i* to its nearest neighbour [23]. The theoretical function, assuming a null landscape generated by a Poisson distribution, was defined as follows and plotted with the CSR envelope estimated by Monte Carlo simulation [23]:


$$G_{pois}(x)=1-exp-c\lambda \;\pi x^{2}$$

If the empirical $\hat{G}(x)$ exceeded the theoretical envelope, leptospirosis cases were considered clustered relative to complete spatial randomness.

#### Polygon-based analysis

Spatial autocorrelation analysis of leptospirosis incidence using the spdep package assessed whether subdistricts with similar incidence rates were geographically clustered, with global autocorrelation assuming uniform incidence across Kelantan. This was quantified by comparing each observation to the mean incidence using Moran’s I [18]:


$$I=\frac{n\Sigma \sum_{1}^{n}W_{ij}(x_{i}-\bar{x})(x_{j}-\bar{x})}{w\Sigma (x_{i}-\bar{x}{)}^{2}}$$

Where *n* is the leptospirosis incidence, $\bar{x}$ is the mean of the variable, *x_i_* is the variable’s value at a particular location *i*, *x_j_* is the variable’s value at another location *j*, and *W_ij_* is a weight indexing location of *i* relative to *j*. A value of 0 indicates no pattern of spatial correlation, while values closer to 1 or -1 indicate stronger spatial autocorrelation (similar values close together) or spatial dispersion (dissimilar values close together), respectively [18]. Spatial relationships were defined using the Queen contiguity approach, where subdistricts were considered neighbours if they shared an edge or vertex. This method captures broader spatial interactions essential for epidemiological analyses, given that disease transmission and environmental exposures are not strictly limited to administrative boundaries. A spatial weight matrix was constructed with row-standardised weights for proportional contributions. As global Moran’s I does not identify local clusters, local Moran’s I, through local indicators of spatial autocorrelation (LISA), was employed to detect regional patterns and outliers. High positive values indicated clustering of similar incidence rates [24,25]. LISA analysis classified spatial clusters as high-high (high incidence in a high-incidence neighbourhood) or low-low (low incidence in a low-incidence neighbourhood), and spatial outliers as high-low (high incidence in a low-incidence neighbourhood) or low-high (low incidence in a high-incidence neighbourhood). Significance was assessed through Monte Carlo permutations at 95% confidence (p<0.05) [25].

### Ethics statement

The study was registered with the National Medical Research Register (NMRR ID-22-02749-XAA), adhering to the Helsinki Declaration and Malaysia Good Clinical Practice Guidelines. Ethical approval was obtained from the Human Research and Ethics Committee of Universiti Sains Malaysia (JEPeM) (reference: USM/JEPeM/22110718) and the Medical Research and Ethics Committee (MREC) of the National Institute of Health.

## RESULTS

### Study characteristics

A total of 1,534 laboratory-confirmed leptospirosis cases notified to the e-Notifikasi online database from 2016 to 2023 were analysed. Leptospirosis cases predominantly affected adults (58.7%) and adolescents (18.3%), with an overall mean age of 31.6 years (95% CI, 30.6 to 32.6) (Table 1). Notably, the proportion of adolescent cases increased from 14.6% in 2022 to 20.1% in 2023, indicating a rise in infections among younger individuals. Males were significantly more affected than females (67.1 vs. 32.9%; *χ*^2^(1): 158.533; p<0.001). Malay ethnicity constituted the majority of cases (≥85% annually), consistent with Kelantan’s demographic profile. Outbreak-associated cases were reported only in 2016, with no further outbreaks observed from 2017 onwards. Intensive care unit admissions peaked in 2023 (21 cases, 4.9%), while outpatient cases increased steadily from 42.7% in 2016 to 72.7% in 2023.

### Spatial distribution of leptospirosis cases

**Figure 1.**
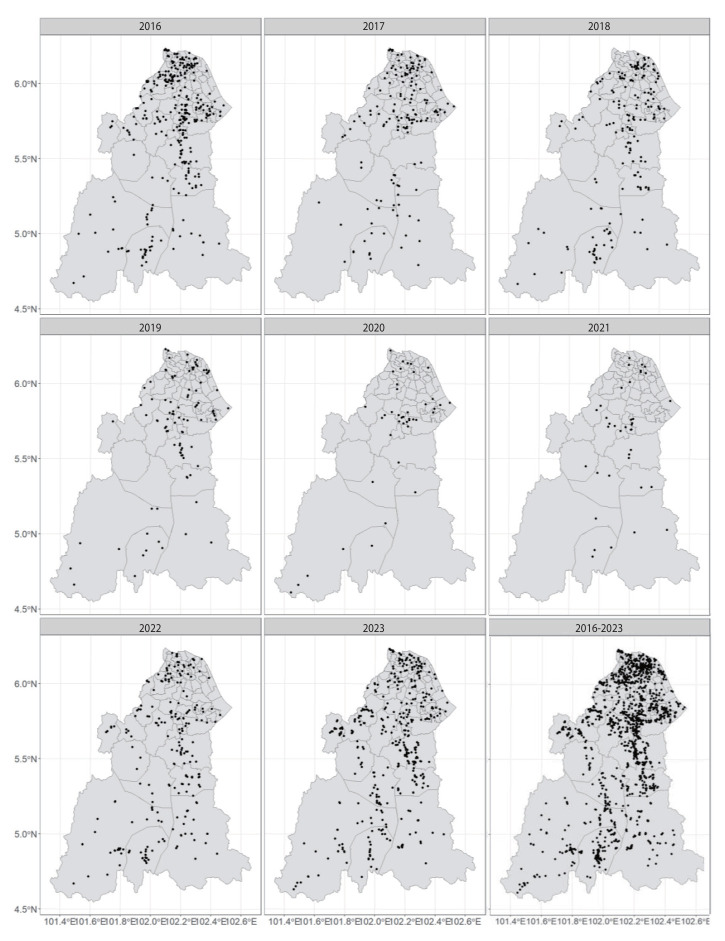
Distribution of laboratory-confirmed leptospirosis cases in Kelantan based on registration year, 2016-2023. Overall, the case distribution is higher in the northern region.

Spatial analysis of laboratory-confirmed leptospirosis cases in Kelantan revealed distinct regional and temporal distribution patterns. All 66 subdistricts reported at least 1 laboratory-confirmed leptospirosis case between 2016 and 2023: 58 subdistricts reported cases in 2016, 57 in 2017, 51 in 2018, followed by a decline to 44 and 32 subdistricts during the pandemic years 2020 and 2021, respectively, before increasing again to 48 subdistricts in 2022 and 58 in 2023 (Figure 1). Between 2016 and 2017, cases were predominantly concentrated in northern and central regions, with fewer cases in the south. This spatial distribution persisted into 2018-2019, with a slight increase observed in the south. A resurgence in cases emerged in 2022, notably in central and southern regions. By 2023, the southern districts exhibited a marked increase in case density, whereas the northern and central regions continued reporting similar case numbers as in previous years.

### Incidence of leptospirosis

**Figure 2.**
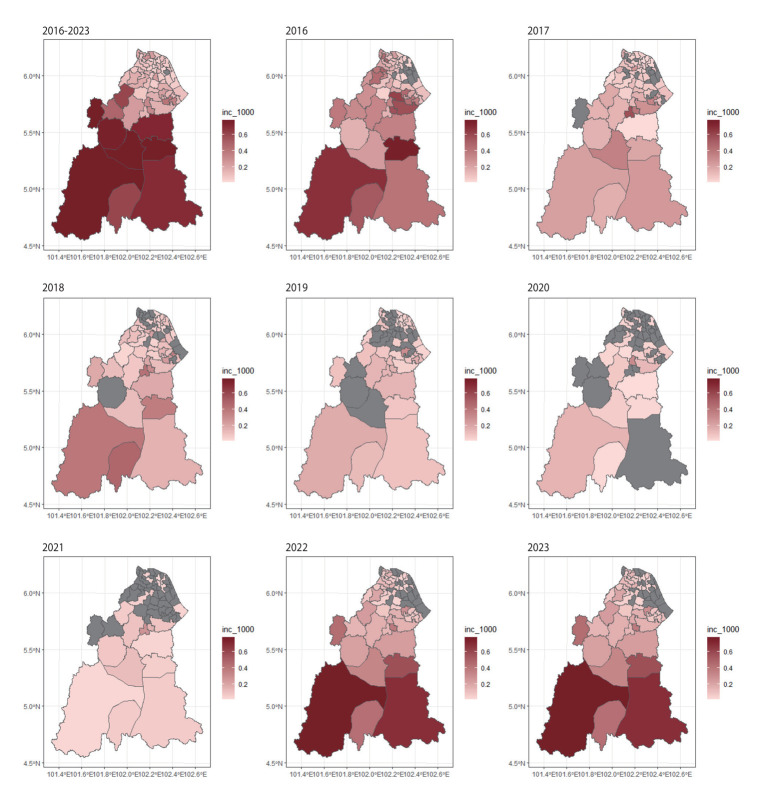
Annual incidence of leptospirosis in Kelantan (2016-2023). Darker shades indicate higher incidence, lighter shades represent lower incidence, and grey indicates no laboratory-confirmed cases registered for that year.

The average annual incidence of leptospirosis between 2016 and 2023 was 0.10 per 1,000 population (95% CI, 0.04 to 0.16), with the highest incidence occurring in the southern region. Incidence rates were significantly higher among males (0.13 per 1,000 population; 95% CI, 0.05 to 0.21) than females (0.07 per 1,000 population; 95% CI, 0.02 to 0.11; *χ*^2^(1)=14.892; p<0.001). High regional variability in incidence rates was noted at both district and subdistrict levels from 2016 to 2023 (Figure 2). In 2016, incidence rates were moderate across Kelantan, with most districts reporting rates below 1.0 per 1,000 population. A similar incidence pattern continued in 2017, without notable high-incidence concentrations. From 2018 to 2019, incidence slightly increased in the southern districts but decreased in the north. Data for 2020-2021 were incomplete, as indicated by grey shading for most districts. However, in 2022, incidence increased notably, surpassing 1.0 per 1,000 population in several areas, particularly southern districts. This increasing trend persisted in 2023, with the highest incidences observed in southern and south-eastern districts, some exceeding 1.5 per 1,000 population. These districts (Gua Musang, Kuala Krai, and Machang) also recorded the highest absolute case counts, indicating that observed clustering was genuinely reflective of increased disease burden rather than artefactual due to low population density.

### Hotspot analysis of leptospirosis cases

**Figure 3.**
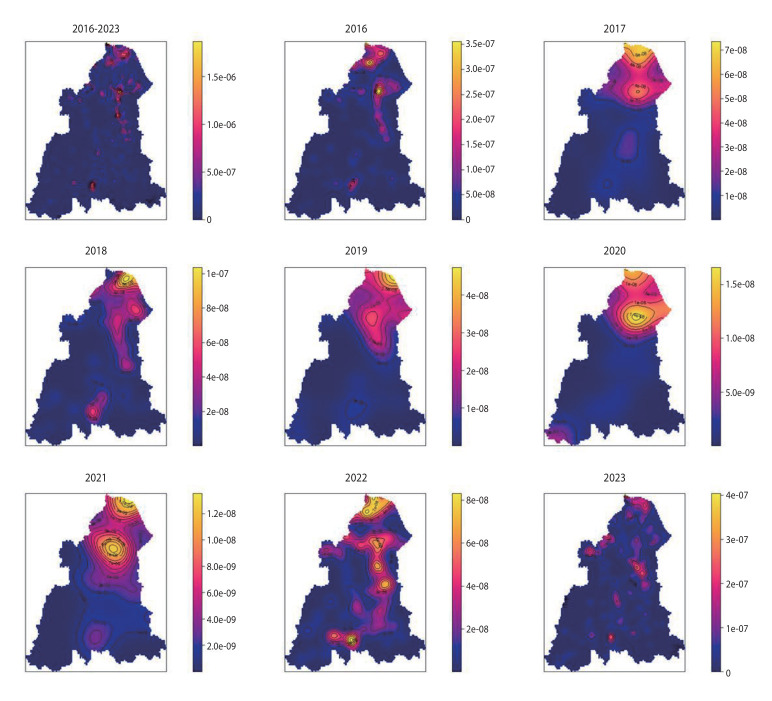
Kernel density estimation of leptospirosis cases in Kelantan (2016-2023). The *y*-axis in the plot represents the estimated density of leptospirosis cases at each location, with higher values indicating a higher concentration of cases and lower values reflecting fewer cases or lower density in that area. Lighter shades indicate higher density, while darker shades represent lower density. Overall, the incidence is higher in the central and southern regions.

KDE analysis revealed distinct changes in leptospirosis hotspots between 2016 and 2023 (Figure 3). From 2016-2018, hotspots primarily concentrated in the northern region, displaying stable, localised distributions. In 2019, hotspot intensity increased, extending spatially towards central regions. Hotspot intensity and spatial extent significantly declined in 2020-2021, reflecting reduced high-risk locations during the coronavirus disease 2019 (COVID-19) pandemic. In 2022, emerging hotspots appeared in the southern and central regions of Kelantan, initially presenting moderate intensity. By 2023, hotspot intensity and spatial spread notably increased in the central region, resulting in an expanded high-transmission area. Meanwhile, southern-region hotspots maintained limited spatial expansion but remained distinctly localised, indicating persistent localised transmission.

### Nearest neighbour analyses of leptospirosis cases

**Figure 4.**
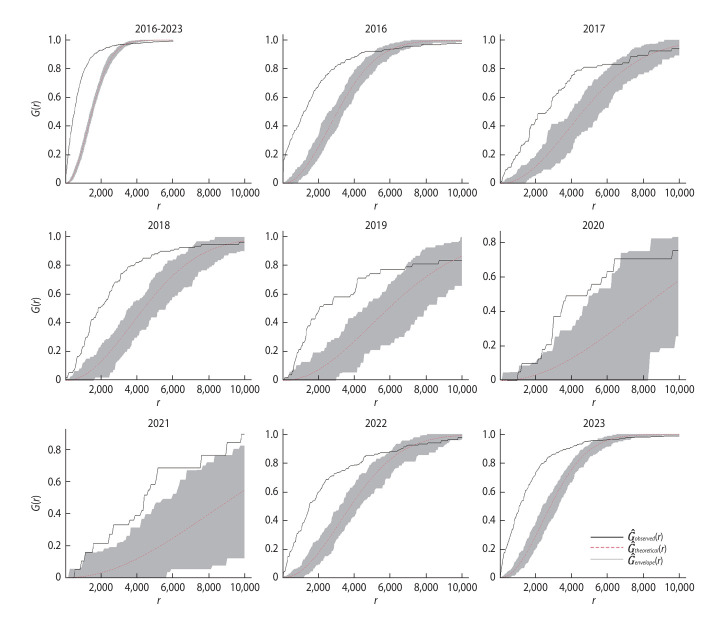
Nearest neighbour distance analysis using the *G*-function under complete spatial randomness for leptospirosis cases in Kelantan, 2016-2023, $\hat{G}$ is denoted by the black line and ${\hat{G}}_{pois}$ by the red dotted line. Values of $\hat{G}>{\hat{G}}_{pois}(r)$ suggest clustered patterns. Significance was tested using the Monte Carlo test based on envelopes of the *G*-function. The grey envelope in the plot signifies the 95% significance level. Overall, the *G*-function showed significant clustering within approximately 4,000 metres.

Nearest neighbour analyses demonstrated significant temporal variations in spatial clustering and dispersion. Strong clustering was observed from 2016 to 2019, indicated by NNI values <1, strong negative Z-scores, and significant p-values (p<0.001; Table 2). Clustering declined in 2020-2021, with NNI values nearing 1, indicative of a more random distribution. Clustering re-emerged in 2022-2023, showing NNI values <1 and large negative Z-scores, reflecting localised hotspots and spill over effects into surrounding areas. The $\hat{G}$-function analysis further detailed variations in clustering scale (Figure 4). On the *y*-axis, the probability of finding the nearest neighbouring case within a distance of *r* metres (*x*-axis) is shown; values closer to 1 signify strong clustering at smaller scales, while values near 0 indicate dispersed patterns. In 2016, clustering appeared at shorter distances (approximately 4,000 m), possibly reflecting specific environmental conditions or localised transmission sources. The range extended to approximately 6,000 m in 2017-2018 and reached approximately 7,000 m by 2019, suggesting gradual spatial expansion. During 2020-2021, clustering dispersed over broader geographical ranges. In 2022-2023, clustering was observed over both short and moderate distances, indicative of localised hotspots combined with broader spatial spread.

### Spatial autocorrelation analysis of leptospirosis incidence

**Figure 5.**
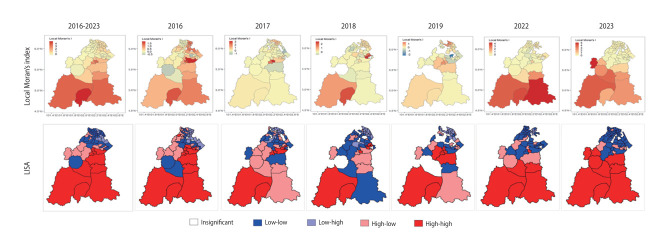
Local Moran’s I Index and local indicator of spatial association (LISA) map depicting spatial clusters and outliers of leptospirosis cases in Kelantan between 2016 to 2023 (excluding the years 2020-2021 due to insignificant autocorrelation).

Leptospirosis incidence displayed significant positive spatial autocorrelation in all years except during the 2020-2021 COVID-19 pandemic period. Global Moran’s I fluctuated, showing a declining trend initially, then increasing again in recent years, reflecting strengthening spatial autocorrelation (Table 2). Local autocor-relation analysis from 2016 to 2023 (excluding 2020-2021 due to insignificant global autocorrelation) identified distinct geographical patterns (Figure 5). The southern region consistently exhibited localised hotspots with high positive Moran’s I-values, forming high-high clusters. Conversely, the northern region predominantly demonstrated low-low clusters, expanding over time and interspersed with spatial outliers (low-high and high-low), accompanied by declining Moran’s I-values indicative of weakening correlations or dispersion. The central region initially showed outliers reflecting local variability in leptospirosis transmission, which later evolved into significant high-high clusters, indicating increasing disease intensity and geographic spread. Additionally, decreasing areas classified as insignificant throughout the study period suggested more robust and defined spatial clustering in recent years. High-high clusters corresponded mainly to subdistricts with high absolute case counts and moderate population densities, implying that observed spatial clusters represented genuine elevations in disease burden rather than being artefacts resulting from small population denominators.

## DISCUSSION

Leptospirosis remains endemic in Malaysia, with continuing high incidence rates in Kelantan and neighbouring states such as Perak, Selangor, and Pahang [26]. Kelantan faces heightened risk for infectious diseases, particularly those related to water, due to heavy rainfall during monsoon seasons that frequently results in flooding. Furthermore, Kelantan has relatively low piped water supply coverage at 67.8%, causing residents to rely heavily on untreated water sources, including dug wells, tube wells, gravitational feed systems, and rivers [11,27]. The leptospirosis case fatality rate in Kelantan was comparable to the national average, at approximately 1.47%. This study found a substantial proportion of leptospirosis cases in adults, with a higher incidence among the elderly, raising concerns about increased morbidity and mortality associated with advanced age [28]. Despite Kelantan having a nearly balanced male-to-female ratio of 1.00:1.05, this study observed leptospirosis predominantly affecting males, likely due to differences in exposure risk. Male typically experience greater exposure to contaminated environments due to occupational activities, whereas sex differences in infection rates become negligible when exposure levels are similar [28]. Additionally, females generally demonstrate better hygiene knowledge, awareness, and practices than males [29].

This study aligns with previous research highlighting the northern region of Kelantan as a consistent leptospirosis hotspot [30]. The elevated number of cases in the northern region is partly attributable to higher population density. However, certain areas within this region exhibited moderate-to-high incidence and high case densities, suggesting that factors beyond population size also play critical roles. In densely populated areas, increased transmission risk is coupled with greater strain on healthcare resources. The northern region remains high-risk for leptospirosis due to inadequate hygiene, waste management challenges, and frequent flooding events, which collectively promote disease transmission [26]. Rapid urbanisation in these areas exacerbates issues related to insufficient sanitation and drainage, creating optimal breeding conditions for *Leptospira* bacteria and increasing human exposure risks. Before the COVID-19 pandemic, case numbers and incidence in these areas had declined, potentially due to improved economic conditions, greater public awareness, better health-seeking behaviour, and enhanced healthcare accessibility in urban settings [11,12]. Conversely, southern districts exhibit lower economic status, higher proportions of immigrants and Indigenous populations, and limited healthcare access, with some settlements near forests and mountainous terrain isolated from essential services such as hospitals and schools [31,32]. Better healthcare access and infrastructure in the north may have improved detection and reporting, potentially leading to a higher number of documented cases.

While the northern region remained affected throughout the study period, new hotspots emerged in central and southern Kelantan. These predominantly rural areas are characterised by expanding agricultural activities, logging operations, palm oil plantations, and nature-based tourism [31,33]. High environmental exposure due to frequent contact with contaminated water, soil, and animals significantly elevates leptospirosis risk in agricultural and forested settings [34,35]. Between 2000 and 2015, approximately 67% of Environmental Impact Assessment projects approved in Kelantan were located in Gua Musang, predominantly related to agriculture and logging, significantly impacting forests, reserves, and watersheds. Such environmental changes likely facilitated increased rodent populations and contamination, accelerating disease transmission [36]. During 2020-2021, a marked reduction in laboratory-confirmed cases occurred, likely due to incomplete reporting, disruptions in healthcare services during the COVID-19 pandemic, altered health-seeking behaviours, quarantine avoidance, vague symptomatology, limited screening, low clinical suspicion, containment measures, economic downturns, and travel restrictions [37,38]. The post-pandemic resurgence of cases likely resulted from increased recreational activities in central and southern regions, where tourism sites are abundant, and a return to pre-pandemic behaviour patterns, elevating transmission risk [39,40].

This study identified significant spatial autocorrelation in leptospirosis incidence, consistent with observations documented in various regions [41,42]. Leptospirosis occurrence is strongly influenced by environmental, socioeconomic, and behavioural factors, resulting in autocorrelated areas that share similar conditions and disparities. Human mobility, such as migration and commuting, further exacerbates spatial clustering [43]. Variations in healthcare accessibility and reporting standards may also introduce bias, as enhanced surveillance and diagnostic capacities increase case detection, potentially generating high-low or low-high clusters despite similar underlying disease burdens [44]. Recognising these factors is crucial for designing region-specific interventions. The observed transition from high-low to high-high clusters highlights the potential for localised outbreaks to evolve into persistent hotspots. Therefore, early identification of high-low clusters can serve as a timely warning for intervention, preventing cluster escalation.

This study observed leptospirosis case clustering at both short and moderate distances, reflecting dynamic interactions between localised transmission and broader spill over effects. Hotspots likely emerge due to increased human activity, including high population density, recreational activities, or occupational practices in high-risk environments [26,28,45]. Environmental changes, such as intensified flooding in northern regions and altered land use practices in southern areas, have likely contributed to the observed expansion of spatial clusters, a trend reported elsewhere [45]. Understanding these patterns informs targeted interventions ranging from improving local sanitation practices to broader public awareness campaigns. Such insights enable optimised resource allocation by focusing efforts in areas where spill over risks could potentially expand outbreaks beyond initial locations.

The study’s findings underscore the importance of analysing both leptospirosis case distribution and incidence patterns in Kelantan. Although the northern region lacked pronounced spatial clustering relative to its neighbouring areas, density analyses based on individual case distributions (rather than population-adjusted rates) revealed persistently high case densities in certain northern subdistricts, potentially reflecting population density and enhanced reporting capacities. Conversely, the southern region displayed consistent high-high clusters, indicating stable hotspots. Case-density analysis provides detailed insights into localised transmission risks, which population-aggregated incidence data may obscure. Absolute case counts and density analyses inform prioritisation of surveillance, resource allocation, and control strategies. Surveillance typically flags high-density regions with substantial case counts as hotspots, while population-based incidence analyses standardise disease burden for regional comparison. Importantly, this study also detected spatial clustering based on incidence rates even in areas not identified as high-density zones through kernel density analysis. Incidence-based analysis accounts for both case numbers and population size, enabling identification of high-risk areas even when absolute case counts are low. Integrating both case distribution and incidence data enhances understanding of regional leptospirosis heterogeneity, essential for targeted interventions. Identifying spatial clustering patterns and transmission distances supports strategic decisions about optimal health facility placement, high-risk population targeting, and timing for outbreak mitigation efforts.

This study has several limitations. First, it relied on passive surveillance data obtained from the e-Notifikasi system, which may result in underreporting and exclusion of non-laboratory-confirmed cases. Additionally, the use of crude incidence rates, necessitated by the absence of age-stratified population data at the subdistrict level, limited the ability to calculate reliable standardised incidence rates. Therefore, observed variations in incidence may reflect differences in population composition rather than actual differences in disease risk. In areas with low population density, crude incidence rates might appear disproportionately high due to small denominators, despite relatively few case counts; thus, while helpful for surveillance purposes, interpretation should carefully consider the underlying population distribution. Furthermore, data limitations restricted this analysis to spatial methods, excluding potentially influential socio-demographic and environmental factors. Future research employing spatial regression analyses could explore critical risk factors such as water sources, waste management practices, land-use patterns, and socioeconomic status. Integrating spatial analyses with real-time epidemiological surveillance and timely dissemination of findings remains essential for effective disease management.

This study offers crucial insights into the spatial trends of leptospirosis in Kelantan, Malaysia, between 2016-2023, revealing increasing incidence and case density, and significant clustering patterns in central and southern regions. Spatial autocorrelation and hotspot trends illustrate the importance of understanding localised transmission patterns and potential cluster expansion. This study highlighted potential socioeconomic and environmental risk factors that contribute towards the pattern. Despite limitations, the findings provide a foundation for future research. Integrating socioeconomic factors into spatial regression models would improve understanding of disease transmission. Targeted region-specific public health initiatives and enhanced surveillance systems are critical in reducing the leptospirosis burden in Kelantan and other similar regions, providing timely and effective disease prevention.

## Figures and Tables

**Figure f6-epih-47-e2025030:**
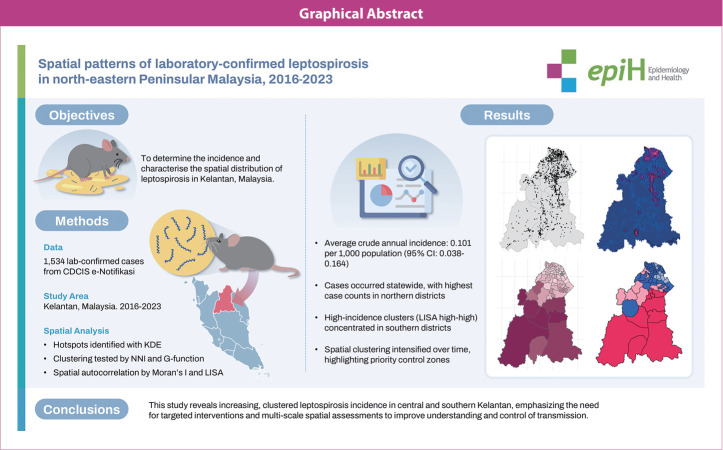


**Table 1.** Characteristics of laboratory-confirmed leptospirosis cases in Kelantan between 2016 and 2023

Characteristics	2016 (n=361)	2017 (n=158)	2018 (n=177)	2019 (n=97)	2020 (n=42)	2021 (n=38)	2022 (n=233)	2023 (n=428)	Overall (n=1,534)
Age, mean±SD (yr)	29.1±19.9	27.4±18.3	30.6±19.7	28.1±20.1	37.8±18.0	35.7±20.4	35.7±18.3	33.3±19.4	31.6±19.5
Children (<-5)	44 (12.2)	13 (8.2)	18 (10.2)	15 (15.5)	1 (2.4)	1 (2.6)	7 (3.0)	14 (3.3)	113 (7.4)
Children (6-9)	20 (5.54)	10 (6.3)	15 (8.47)	5 (5.2)	2 (4.8)	1 (2.6)	3 (1.3)	33 (7.7)	89 (5.8)
Adolescent (10-19)	74 (20.5)	38 (24.1)	22 (12.4)	17 (17.5)	3 (7.1)	7 (18.4)	34 (14.6)	86 (20.1)	281 (18.3)
Adult (20-64)	188 (52.1)	89 (56.3)	107 (60.5)	50 (51.5)	32 (76.2)	21 (55.3)	164 (70.4)	250 (58.4)	901 (58.7)
Older adult (≥65)	35 (9.7)	8 (5.1)	15 (8.5)	10 (10.3)	4 (9.5)	8 (21.1)	25 (10.7)	45 (10.5)	150 (9.8)
Sex									
Female	136 (37.7)	51 (32.3)	58 (32.8)	29 (29.9)	17 (40.5)	11 (28.9)	65 (27.9)	137 (32.0)	504 (32.9)
Male	225 (62.3)	107 (67.7)	119 (67.2)	68 (70.1)	25 (59.5)	27 (71.1)	168 (72.1)	291 (68.0)	1,030 (67.1)
Race									
Malay	329 (91.1)	150 (94.9)	162 (91.5)	92 (94.8)	39 (92.9)	36 (94.7)	208 (89.3)	366 (85.5)	1,382 (90.1)
Aborigines	9 (2.5)	3 (1.9)	3 (1.7)	4 (4.1)	1 (2.4)	0 (0)	7 (30.0)	22 (5.1)	49 (3.2)
Chinese	4 (1.1)	0 (0)	2 (1.1)	0 (0)	0 (0)	0 (0)	3 (1.3)	3 (0.7)	12 (0.8)
Indian	0 (0)	0 (0)	3 (1.7)	1 (13.0)	0 (0)	0 (0)	1 (0.4)	3 (0.7)	8 (0.4)
Others	1 (0.3)	0 (0)	0 (0)	0 (0)	0 (0)	0 (0)	3 (1.3)	5 (1.2)	9 (0.4)
Foreigner	18 (5.0)	5 (3.2)	7 (4.0)	0 (0)	2 (4.7)	2 (5.3)	11 (4.7)	29 (6.8)	74 (4.8)
Case classification									
Outbreak	5 (1.4)	0 (0)	0 (0)	0 (0)	0 (0)	0 (0)	0 (0)	0 (0)	46 (3.0)
Sporadic	356 (98.6)	158 (100)	177 (100)	97 (100)	42 (100)	38 (100)	233 (100)	428 (100)	1,488 (97.0)
Hospitalization									
Intensive care unit	12 (3.3)	6 (3.8)	10 (5.7)	7 (7.2)	5 (11.9)	2 (5.3)	16 (6.8)	21 (4.9)	79 (5.2)
General ward	195 (54.0)	74 (46.8)	91 (51.4)	40 (41.2)	19 (45.2)	16 (42.1)	88 (37.8)	96 (22.4)	619 (40.3)
Outpatient	154 (42.7)	78 (49.4)	76 (42.9)	50 (51.5)	18 (42.9)	20 (52.6)	129 (55.4)	311 (72.7)	836 (54.5)
Case outcome									
Alive	358 (99.2)	158 (100)	177 (100)	96 (99.0)	42 (100)	36 (94.7)	225 (96.6)	424 (99.1)	1,516 (98.8)
Dead	3 (0.8)	0 (0)	0 (0)	1 (1.0)	0 (0)	2 (5.3)	8 (3.4)	4 (0.9)	18 (1.2)
District									
Bachok	1 (0.3)	3 (1.9)	12 (6.8)	6 (6.2)	1 (2.4)	0 (0)	1 (0.4)	6 (1.4)	30 (2.0)
Gua Musang	60 (16.6)	20 (12.7)	41 (23.2)	15 (15.5)	6 (14.3)	6 (15.8)	72 (30.9)	91 (21.3)	311 (20.3)
Jeli	16 (4.4)	6 (3.8)	5 (2.8)	1 (1.0)	0 (0)	1 (2.6)	12 (5.2)	46 (10.7)	87 (5.7)
Kota Bharu	25 (6.9)	26 (16.5)	30 (16.9)	16 (16.5)	2 (4.8)	6 (15.8)	16 (6.9)	41 (9.6)	162 (10.6)
Kuala Krai	55 (15.2)	17 (10.8)	30 (16.9)	15 (15.5)	3 (7.1)	7 (18.4)	46 (19.7)	111 (25.9)	284 (18.5)
Machang	52 (14.4)	28 (17.7)	17 (9.6)	8 (8.2)	11 (26.2)	8 (21.1)	19 (8.2)	22 (5.1)	165 (10.8)
Pasir Mas	64 (17.7)	17 (10.8)	14 (7.9)	7 (7.2)	4 (9.5)	1 (2.6)	23 (9.9)	23 (5.4)	153 (10.0)
Pasir Puteh	29 (8.0)	14 (8.9)	17 (9.6)	11 (11.3)	6 (14.3)	1 (2.6)	8 (3.4)	25 (5.8)	111 (7.2)
Tanah Merah	26 (7.2)	16 (10.1)	9 (5.1)	15 (15.5)	6 (14.3)	6 (15.8)	21 (9.0)	36 (8.4)	135 (8.8)
Tumpat	33 (9.1)	11 (7.0)	2 (1.1)	3 (3.1)	3 (7.1)	2 (5.3)	15 (6.4)	27 (6.3)	96 (6.3)

Values are presented as number (%).

**Table 2.** Clustering analysis of leptospirosis incidence in Kelantan (2016-2023) for leptospirosis cases (nearest neighbour analysis) and leptospirosis incidence (global Moran’s I spatial autocorrelation analysis)

Year	ANN (km)	NNI			Global Moran’s I	
		NNI	Z-score	p-value	*I*	p-value
2016	1.793	0.597	-14.632	<0.001	0.451	<0.001
2017	3.282	0.807	-4.643	<0.001	0.156	<0.001
2018	2.792	0.681	-8.116	<0.001	0.274	<0.001
2019	4.385	0.761	-4.511	<0.001	0.210	0.026
2020	7.359	1.043	0.528	0.597	0.096	0.138
2021	6.394	0.950	-0.595	0.552	0.185	0.077
2022	2.534	0.692	-8.999	<0.001	0.419	<0.001
2023	1.608	0.572	-16.920	<0.001	0.549	<0.001

ANN, average nearest neighbour; NNI, nearest neighbour index: >1 indicates dispersion, <1 indicates clustering under complete spatial randomness.

## Supplementary materials

Supplementary Material 1. Peninsular Malaysia map with a focus on Kelantan, displaying district boundaries and labels for spatial reference.

Supplementary Material 2. Demographic Profile of Kelantan Districts Based on the 2020 Census
