# Supplementary material for: Spatial patterns of laboratory-confirmed leptospirosis in north-eastern Peninsular Malaysia, 2016-2023
Source: Epidemiol Health. 2025 May 29;47:e2025030. doi: 10.4178/epih.e2025030 (PMC12425861; doi:10.4178/epih.e2025030)
Supplement: Supplementary Material 1. — Peninsular Malaysia map with a focus on Kelantan, displaying district boundaries and labels for spatial reference. [file epih-47-e2025030-Supplementary-1.pptx]

## Slide 1
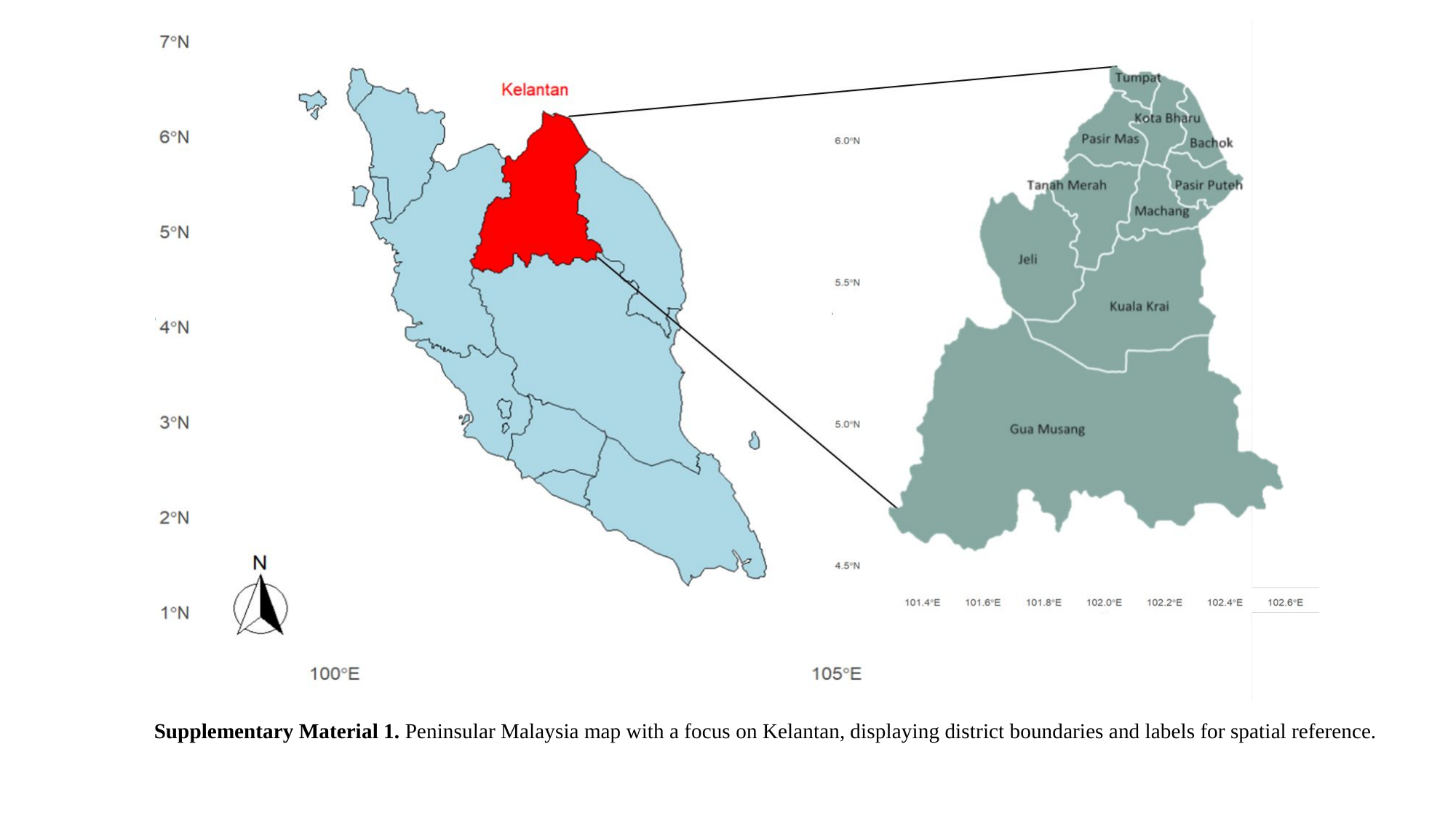

Supplementary Material 1. Peninsular Malaysia map with a focus on Kelantan, displaying district boundaries and labels for spatial reference.
