# Supplementary material for: Spatial patterns of laboratory-confirmed leptospirosis in north-eastern Peninsular Malaysia, 2016-2023
Source: Epidemiol Health. 2025 May 29;47:e2025030. doi: 10.4178/epih.e2025030 (PMC12425861; doi:10.4178/epih.e2025030)
Supplement: Supplementary Material 2. — Demographic Profile of Kelantan Districts Based on the 2020 Census [file epih-47-e2025030-Supplementary-2.docx]

**Supplementary Material 2.** Demographic Profile of Kelantan Districts Based on the 2020 Census

| **Characteristics** | **Bachok** | **Kota Bharu** | **Machang** | **Pasir Mas** | **Pasir Puteh** | **Tanah Merah** | **Tumpat** | **Gua Musang** | **Kuala Krai** | **Jeli** |
| --- | --- | --- | --- | --- | --- | --- | --- | --- | --- | --- |
| Total Population, ‘000 | 10.7 | 568.9 | 115.5 | 233.4 | 138.4 | 153.9 | 183.1 | 113.4 | 106.3 | 56.3 |
| Male, '000 | 5.5 | 284.6 | 57.4 | 118.2 | 68.6 | 79.0 | 90.7 | 54.6 | 53.3 | 28.1 |
| Female, '000 | 5.2 | 284.3 | 58.2 | 115.1 | 69.8 | 74.9 | 92.4 | 48.1 | 52.9 | 28.2 |
| Citizens, '000 (%) | 10.4 (97.6) | 557(97.9) | 114.6(99.2) | 231.1(99.0) | 137.6 (99.4) | 149.5 (97.1) | 181.9(99.3) | 107.3(94.6) | 103.3(97.2) | 55.1(97.7) |
| Non-citizens, '000 (%) | 0.3(2.4) | 11.9(2.1) | 0.9(0.8) | 2.3(1.0) | 0.8(0.6) | 4.5(2.9) | 1.2(0.7) | 6.1(5.4) | 3.0(2.8) | 1.2(2.1) |
| Annual population growth, % | 0.2 | 1.2 | 2.3 | 0.6 | 0.8 | 1.0 | 0.8 | 0.1 | 0.4 | 1.0 |
| Population density, per km^2^ | 6.0 | 1413.0 | 220.0 | 407.0 | 220.0 | 175.0 | 1018.0 | 16.0 | 47.0 | 42.0 |
| Average household size | 4.9 | 4.7 | 5.0 | 5.0 | 5.0 | 4.9 | 5.1 | 4.9 | 4.4 | 4.5 |
| Number of households, '000 | 2.2 | 119.8 | 22.9 | 46.0 | 22.9 | 31.4 | 36.0 | 20.8 | 24.3 | 12.4 |
| Number of living quarters, '000 | 2.7 | 139.8 | 26.6 | 56.4 | 27.3 | 36.4 | 44.4 | 27.0 | 29.9 | 12.0 |
| Population in rural area, % | 55.9 | 8.2 | 76.0 | 78.2 | 82.0 | 62.1 | 64.9 | 69.3 | 74.2 | 100.0 |
| Poverty incidence, % | 13.1 | 9.1 | 7.1 | 13.1 | 12.7 | 12.0 | 12.5 | 17.2 | 22.4 | 18.5 |
| Source: Department of Statistic Malaysia, Data as of MyCencus 2020 [11] | | | | | | | | | | |
